# Erratum

**DOI:** 10.1159/pps/aemag011

**Published:** 2026-09-07

In the article, “Does fatigue in patients with Post-COVID-19 Syndrome improve with rehabilitation?” Rehabilitation. Psychotherapy and Psychosomatics. 2026:1–15. https://doi.org/10.1159/000549848 by Kupferschmitt et al., noted there was an error in Table 4.

**Table 4.** Symptom course – differences within the depressed and nondepressed sample from admission to discharge

​	Depressed PCS patients, *n* = 734 (71.4%)						Nondepressed PCS patients, *n* = 294 (28.6%)					
	admission	discharge	*p* value	d	MWD	CL	admission	discharge	*p* value	d	MWD	CL
	M (SD)	M (SD)					M (SD)	M (SD)				
*PHQ-9 (scale 1–4)*												
Loss of joy or interest	2.91 (0.80)	2.43 (0.66)	<0.001	0.64	−0.48	0.39 to 0.57	1.51 (0.51)	1.34 (0.48)	<0.001	0.35	−0.19	0.09 to 0.25
Dejection/melancholy/hopelessness	2.69 (0.8 24)	2.39 (0.69)	<0.001	0.37	−0.30	0.20 to 0.39	1.14 (0.36)	1.16 (0.38)	0.381	–	0.02	−0.08 to 0.03
Difficulty falling asleep/staying asleep	3.46 (0.78)	3.23 (0.84)	<0.001	0.28	−0.23	0.13 to 0.32	2.81 (1.06)	2.61 (1.07)	0.023	0.18	−0.20	0.02 to 0.37
Tiredness	3.64 (0.63)	3.19 (0.82)	<0.001	0.62	−0.45	0.35 to 0.53	3.13 (0.96)	2.61 (1.01)	<0.001	0.52	−0.52	0.35 to 0.67
Loss of appetite	2.27 (1.06)	2.01 (0.94)	<0.001	0.54	−0.26	0.13 to 0.37	1.55 (0.83)	1.36 (0.63)	0.001	0.26	−0.19	0.07 to 0.31
Self-deprecation and self-doubt	2.24 (1.04)	1.98 (0.93)	<0.001	0.24	−0.26	0.13 to 0.38	1.17 (0.44)	1.16 (0.45)	0.861	–	−0.01	−0.06 to 0.08
Concentration problems	3.26 (0.84)	3.04 (0.89)	<0.001	0.26	−0.22	0.11 to 0.33	2.76 (1.07)	2.37 (0.99)	<0.001	0.38	−0.40	0.22 to 0.56
Psychomotor changes: Restlessness or retardation	2.33 (1.03)	2.08 (0.94)	<0.001	0.24	−0.25	0.10 to 0.39	1.83 (1.03)	1.55 (0.77)	0.001	0.31	−0.28	0.11 to 0.45
Suicidal thoughts	1.30 (0.59)	1.19 (0.51)	<0.001	0.18	−0.11	0.03 to 0.17	1.00 (0.13)	1.00 (0.12)	0.932	–	–	−0.02 to 0.02
PHQ-9 total value	24.18 (4.35)	21.47 (4.31)	<0.001	0.62	−2.71	2.06 to 3.35	16.78 (3.75)	15.23 (3.74)	<0.001	0.41	−1.55	0.81 to 2.29
*FSMC Fatigue (5-point Likert Scale, max 50 points per subscale and 100 points for the overall scale*												
Cognitive fatigue	40.74 (7.01)	40.17 (7.59)	0.2.08	–	−0.57	−0.69 to 2.61	34.79 (9.99)	33.98 (10.48)	0.344	–	−0.81	−0.87 to 2.49
Motoric fatigue	40.09 (6.49)	39.63 (7.04	0.277	–	−0.46	−0.32 to 1.46	34.34 (9.22)	33.20 (9.76)	0.154	–	−1.14	−0.43 to 2.71
FSMC total value	80.77 (12.70)	79.81 (14.12)	0.256	–	−0.96	−0.36 to 1.28	69.09 (18.46)	67.21 (19.53)	0.242	–	7.12	−1.27 to 5.04
*WHODAS2.0 Limitations (5-point Likert Scale)*												
Limitations mobility	2.22 (1.14)	2.13 (1.07)	0.551	–	−0.09	−0.11 to 0.20	2.31 (1.16)	1.28 (0.66)	0.085	–	−0.21	−0.02 to 0.43
Limitations on understanding/communication	3.28 (0.85)	3.03 (0.93)	0.070	–	−0.25	−0.02 to 0.52	1.49 (0.87)	1.83 (1.900	0.036	0.44	−0.48	0.03−0.93
Limitations on self-sufficiency	1.23 (0.59)	1.29 (0.62)	0.278	–	−0.06	−0.16 to 0.04	1.04 (0.27)	1.00 (0.13)	0.065	–	−0.04	0.00 to 0.08
Limitations on dealing with people	2.21 (1.02)	2.07 (1.10)	0.335	–	−0.13	−0.14 to 0.42	1.31 (0.67)	1.12 (0.45)	0.010	0.34	−0.19	0.04 to 0.33
Limitations in everyday life	3.81 (0.91)	2.97 (1.44)	<0.001	0.71	−0.84	0.45 to 1.22	3.03 (1.40)	1.49 (1.02)	<0.001	1.30	−1.54	1.03 to 2.04
Limitations in social life	3.50 (0.78)	3.17 (0.76)	0.881	–	−0.32	−0.28 to 0.24	2.38 (1.05)	1.93 (0.93)	0.040	0.45	−0.45	−0.5 to 0.94

M, mean; SD, standard deviation; p, level of significance; Cohen-d-effect size, 0.2 small, 0.5 medium, 0.8 big.

In Table 4, the error regarding the average FSMC total score in non-depressed subgroup (mistake T1: M = 60.09). The correct values are as follows:

Total fatigue T1 M = 69.09, SD = 18.46; T2 M = 67.21, SD = 19.53.

The error occurred due to typographical errors during the manual extraction of numerical values when the statistical results were generated.

The corrected Table 4 is shown here.

